# Early BCR-ABL1 decline in imatinib-treated patients with chronic myeloid leukemia: results from a multicenter study of the Chinese CML alliance

**DOI:** 10.1038/s41408-018-0093-4

**Published:** 2018-06-15

**Authors:** Jingru Zhang, Yingqiao Wang, Jianxiang Wang, Jianda Hu, Suning Chen, Jie Jin, Ting Liu, Jianfeng Zhou, Yu Hu, Daoxin Ma, Xiaojun Huang, Chunyan Ji, Ming Hou

**Affiliations:** 1 Department of Hematology, Qilu Hospital, Shandong University, Jinan, Shandong China; 2 Institute of Hematology and Blood Diseases Hospital, Chinese Academy of Medical Sciences and Peking Union Medical College, Tianjin, China; 3 Fujian Provincial Key Laboratory of Hematology, Fujian Institute of Hematology, Fujian Medical University Union Hospital, Fuzhou, Fujian China; 4 Jiangsu Institute of Hematology, Key Laboratory of Thrombosis and Hemostasis of Ministry of Health, The First Affiliated Hospital of Soochow University, Suzhou, Jiangsu China; 5 Department of Hematology, The First Affiliated Hospital, Zhejiang University College of Medicine, Hangzhou, Zhejiang, China; 6 Department of Hematology, West China Hospital, Sichuan University, Chengdu, Sichuan China; 7 Department of Hematology, Tongji Hospital, Tongji Medical College, Huazhong University of Science and Technology, Wuhan, Hubei China; 8 Institute of Hematology, Union Hospital, Tongji Medical College, Huazhong University of Science and Technology, Wuhan, Hubei China; 9 Institute of Hematology, Peking University People’s Hospital, Beijing, China

## Abstract

An early molecular response is spectacularly predictive of outcome in chronic myeloid leukemia (CML) and early response landmarks may identify the high-risk patients likely to be benefit from an early therapy switch. In this study, we evaluated the most relevant cutoffs for early molecular response markers (BCR-ABL1 values at 3 months, log reduction and halving time between diagnosis and 3 months) in 476 first-line imatinib-treated Chinese patients with chronic phase CML. All outcomes were significantly superior for the 324 patients with 3-month BCR-ABL1 ≤10%, so did for the 270 patients with BCR-ABL1 >0.61 log reduction. BCR-ABL1 halving time ≤22 days was identified for patients with the most favorable outcome. Moreover, the prognosis was significantly poorest for patients with both halving time >44 days and BCR-ABL1 >10%. Importantly, multivariate regression analysis demonstrated that a BCR-ABL1 log reduction calculated at 3 months of 0.61 was the only variable that significantly predicted for OS. Our results highlight the importance of rapid initial decline of BCR-ABL1 in predicting satisfactory outcome. Our data support the evidence that monitoring BCR-ABL1 values at an early time point could contribute to accurately assess response and ultimately guide clinical decisions regarding the timing of therapeutic intervention.

## Introduction

Prognosis of patients with chronic myeloid leukemia (CML) has been dramatically improved with the introduction of imatinib as the first tyrosine kinase inhibitor (TKI)^1^. Early molecular response (EMR) to TKI treatment has a strong predictive value in CML patients and early response landmarks may identify patients at higher risk for transformation and poor outcome, who would benefit from an early switch to second-line therapy^2–4^. Assessment of BCR-ABL1 at 3 months has been demonstrated to be the only indicator for predicting prognosis^5^. Indeed, BCR-ABL1 transcript level ≤10% on the international reporting scale (IS) at 3 months is consistently associated with significantly superior overall survival (OS), progression-free survival (PFS), event-free survival (EFS), failure-free survival (FFS), as well as cytogenetic and molecular responses^6,7^. Therefore, this molecular milestone value was incorporated into the National Comprehensive Cancer Network (NCCN) and European Leukemia Net (ELN) recommendations for the management of CML^8^.

Although EMR evaluation criteria seem promising, various factors such as baseline biological characteristics, treatment intensity and tolerance may have an impact on the treatment response and survival of CML patients^9^. Specifically, BCR-ABL1 transcript values at diagnosis are markedly different among chronic phase (CP) CML patients^10,11^. Since imatinib-treated newly diagnosed CML patients often require transient discontinuation of treatment during the early phase due to adverse events, treatment responses and outcomes may be underestimated by BCR-ABL1 transcript levels at 3 months^2,3^. A more precise prognosis evaluation method is urgently needed, which reflects individual clinical entities in patients with CML-CP.

Compared to the effect on the worldwide population (IRIS trial)^12,13^, the effect of imatinib in the Chinese subset was relatively favorable while the incidence of non-hematological toxicities seemed to be more frequent, in part because Chinese patients have higher plasma concentrations of imatinib^14,15^. Recent studies proposed that the rate of BCR-ABL1 decline is a critical prognostic predictor in CML treated with TKI and patients at risk of disease progression can be identified precisely by the lack of a half-log reduction of BCR-ABL1 transcripts at 3 months^10,11^. However, there is no evident information predicting treatment response and prognosis especially in the Chinese population, with the distinct clinical profile of TKI therapy.

In this multicenter study, we investigated the prognostic significance of patient baseline characteristics and EMR markers, as well as evaluated the ability of BCR-ABL1 kinetics to predict outcome in our cohort of 476 first-line imatinib-treated CML patients. Additionally, we explored a better early discriminator of the highest-risk/lowest-risk patients at 3 months, which would help to refine recommendations for treatment decisions at early time points.

## Materials and methods

### Patient sample

From April 2000 to May 2016, a total of 693 patients with newly diagnosed CML from nine Chinese hospitals were enrolled in our clinical trials, data entry was closed on 23 November 2016. These trials included a subset of patients from Peking University People’s Hospital (*n* = 197), Tianjin Institute of Hematology and Blood Diseases Hospital (*n* = 96), Fujian Medical University Union Hospital (*n* = 96), The First Affiliated Hospital of Soochow University (*n* = 80), The First Affiliated Hospital of Zhejiang University (*n* = 73), Qilu Hospital of Shandong University (*n* = 70), West China Hospital of Sichuan University (*n* = 44), Tongji Hospital of Huazhong University of Science and Technology (*n* = 23), and Union Hospital of Huazhong University of Science and Technology (*n* = 14). Only CML-CP patients who were ≥14 years old and expressing typical BCR-ABL1 transcripts (b2a2, b3a2, or b2a2 and b3a2) were included. Besides, patients with imatinib onset before baseline sample collection as well as patients pretreated with hydroxyurea of more than 6 months were excluded from the analysis. A total of 476 imatinib-treated patients were investigated. BCR-ABL1 transcript levels at diagnosis and 3 months were available in 412 patients for review and were thereafter included in this study for final analysis. Written informed consent was obtained from each patient. This study was approved by the ethics committee at each participating institution and was conducted in accordance with the Declaration of Helsinki.

### Molecular analysis

The expression levels of BCR-ABL1 transcripts were determined by quantitative real-time PCR as described previously^16^. ABL1 as the control gene has been studied extensively for suitability for BCR-ABL1 measurement^5^. Results were presented as *BCR-ABL1*/*ABL1*%^IS^, with each participating laboratory-specific values converted to IS. Molecular monitoring was performed prior to commencing imatinib (baseline), at 3, 6, and 12 months, and every 3–6 months thereafter.

### Assessment of treatment response

Cytogenetic and molecular responses were defined according to European Leukemia Net criteria^8^. Cytogenetic assessment was performed at baseline, 3, 6, and 12 months, and every 12 months after the achievement of complete cytogenetic remission (CCyR). If the optimal response has not been achieved, cytogenetic monitoring should be conducted every 3–6 months. The achievement of a major molecular response (MMR ≤0.10%^IS^) and molecular response 4.5 (MR^4.5^ ≤0.0032%^IS^) required confirmation at two consecutive measurements. OS was defined by absence of death from any reason, PFS by absence of accelerated phase (AP), blast crisis (BC) and death from any reason. EFS was measured from the start of treatment to the date of any of the following events while on therapy: loss of complete hematologic remission (CHR), loss of major cytogenetic remission (MCyR), progression to AP/BC or death from any cause^17^. Because of the limitations of this definition, we also measured FFS that accounts for other events such as lack of milestone responses at 3, 6, and 12 months, loss of cytogenetic or molecular response, acquisition of BCR-ABL1 mutations, clonal chromosomal abnormalities in Ph^+^ cells, intolerance, or treatment discontinuation for any reason^11^.

### Log reduction of BCR-ABL1 transcript and halving time calculation

The rate of BCR-ABL1 decline from baseline was assessed by estimating the log reduction and halving time of BCR-ABL1 values. The log reduction in BCR-ABL1 transcript level was defined as log (transcript level at diagnosis/transcript level at 3 months). The halving time defines the number of days over which the BCR-ABL1 transcripts achieve one-half of the baseline value, and was calculated as ln2 × c/[ln(a) − ln(b)] where (a) is the transcript value at diagnosis, (b) the transcript value of the 3-month follow-up and (c) the number of days between both measurements. Where there was no BCR-ABL1 decline from baseline at 3 months, halving times were negative value (*n* = 41 in our study). To enable assessment of the discriminatory power of halving time, the halving times of those patients were imputed to the longest positive halving time of 8000 days, which was calculated for the patient with the smallest reduction^11^.

### Statistical analysis

Receiver operating characteristic (ROC) curves were generated and area under the curve (AUC) was used to compare the correlations between factors and prognosis. The optimal thresholds along the ROC curves were determined using the Youden index. Survival probabilities were performed using the Kaplan–Meier method and compared by the log-rank test. Hazard ratios (HR) and 95% confidential interval (CI) were derived using the Cox proportional hazard model. Cumulative incidence probability curves were calculated to analyze treatment response considering all permanent discontinuations of TKI for any reason as competing risks. Relative risks (RR) were calculated from Fine–Gray regression models, and significance was determined with the Wald test. Level of significance was 0.05. All calculations were performed with the SPSS software Version 13.0.

## Results

### Patient characteristics and outcomes

Patient demographics and disease characteristics at the time of imatinib therapy are presented in Table 1. A total of 476 patients with a median age of 39 years were analyzed. The median follow-up duration of imatinib therapy was 34 months (range 2–126). Disease progression was observed in 24 patients (5.0%), 16 of them died (3.4%).

**Table 1:** Patient characteristics (*n* = 476)

	*N* (%)
Age	39 years (range 14–87)
Sex, male, *N* (%)	301 (63%)
Sokal risk, *N* (%) Low/INT/High/NA	156(33)/193(41)/87(18)/40(8)
Interval since diagnosis, months, median (range)	0.3 (0–6)
MDDs of imatinib (mg/day), median (range)	400 (112.6–748.7)
MDDs of imatinib (mg/day), *n* (%)	
≥100 to <400	125 (26%)
400	304 (64%)
>400 to ≤800	47 (10%)
Baseline *BCR-ABL1*^*IS*^ transcript (%), median (range) (NA = 61)	43.20 (0.0114–598.64)
3-month *BCR-ABL1*^*IS*^ transcript (%), median (range) (NA = 3)	3.55 (0.0025–141.62)

*NA* not available, *INT* intermediate, *MDDs* mean daily doses

With regard to prognostic significance, survival analysis showed that age and gender had no detectable effect on clinical outcome and achievement of molecular response (Table 2). The mean daily doses (MDDs) of imatinib led to differences in the percentage of patients with ≤10% BCR-ABL1 at 3 months. Of the 348 evaluable patients with ≥the median MDDs of 400 mg, 249 (71.6%) had BCR-ABL1 values ≤10% at 3 months. In contrast, 74 of 125 (59.2%) of patients treated with <400 mg had BCR-ABL1 values ≤10% at 3 months. However, no MDDs cutoff could be identified that allowed a discrimination concerning OS, PFS, EFS, or PFS, as well as no statistical difference in outcome was found among the dose groups (Table 2). Therefore, treatment dose was not considered as a prognostic factor for further analysis.

**Table 2:** Probabilities of OS, PFS, EFS, and PFS at 3 years as well as CCyR at 1 years, MMR at 1.5 years and MR4.5 at 3 years by univariate analysis of baseline variables and the *BCR-ABL1* value, log reduction, halving time at 3 months

Variable	No.		OS (%)	Hazard ratio	*P* value	PFS (%)	Hazard ratio	*P* value	EFS (%)	Hazard ratio	*P* value	FFS (%)	Hazard ratio	*P* value	CCyR (%)	Relative risk	*P* value	MMR (%)	Relative risk	*P* value	MR^4.5^ (%)	Relative risk	*P* value
Age (median years)																							
≤39	215	97.7		1	0.239	94.9	1	0.847	92.1	1	0.991	66.5	1	0.575	73.0	1	0.146	56.3	1	0.136	24.2	1	0.594
>39	200	95.5		1.927		94.5	1.071		92.0	1.004		69.5	0.907		66.5	0.842		50.0	0.817		27.0	1.109	
Sex, *n* (%)																							
Female	157	98.7		1	0.083	94.9	1	0.839	92.4	1	0.766	70.7	1	0.21	67.5	1	0.320	55.4	1	0.961	27.4	1	0.989
Male	258	95.3		3.752		94.6	1.094		91.9	1.114		66.3	1.257		71.3	1.129		51.9	0.993		24.4	0.997	
MDDs (mg/day), *n* (%)																							
≥100 to <400	117	95.7		1	0.721	93.2	1	0.611	90.6	1	0.802	55.6	1	0.015	61.5	1	0.155	52.1	1	0.096	27.4	1	0.272
400	258	97.3		0.844		95.7	0.839		93.0	0.849		74.0	0.739		74.0	1.073		55.0	0.986		25.2	0.99	
>400 to ≤800	40	95.0		1.051		92.5	1.036		90.0	1.033		65.0	0.810		67.5	1.463		45.0	1.391		22.5	1.287	
Baseline BCR/ABL1^IS^ transcript																							
≤43%	207	95.7		1	0.341	93.2	1	0.261	89.9	1	0.160	63.3	1	0.123	74.4	1	0.628	55.1	1	0.252	27.1	1	0.994
>43%	208	97.6		0.588		96.2	0.608		94.2	0.601		72.8	0.763		65.4	0.767		51.4	0.857		24.0	1.001	
3-month BCR/ABL1^IS^ transcript																							
≤10%	281	98.2		1	0.012	97.5	1	0.001	95.0	1	0.001	80.1	1	0.001	81.5	1	0.001	66.5	1	0.001	35.6	1	0.001
>10%	131	93.1		4.045		88.5	4.881		85.5	3.104		42.7	4.089		45.0	0.354		24.4	0.269		4.6	0.134	
Log reduction																							
>0.61	270	98.5		1	0.007	97.8	1	0.001	95.6	1	0.001	82.6	1	0.001	79.3	1	0.001	66.3	1	0.001	34.8	1	0.001
≤0.61	142	93.0		4.894		88.7	5.245		85.2	3.460		40.8	4.720		52.1	0.475		28.2	0.347		8.5	0.270	
Halving time																							
≤22 days	166	98.8		1	0.036	98.2	1	0.016	97.0	1	0.004	84.9	1	0.001	86.1	1	0.001	78.3	1	0.001	45.2	1	0.001
>22 days	246	95.1		4.203		92.3	4.458		88.6	4.010		56.9	3.646		64.2	0.435		36.2	0.323		12.6	0.260	

*OS* overall survival, *PFS* progression-free survival, *EFS* event-free survival, *FFS* failure-free survival, *CCyR* complete cytogenetic remission, *MMR* major molecular response, *MR*^*4.5*^ molecular response 4.5, *MDDs* mean daily doses

### Predictive impact of BCR-ABL1 transcript levels at diagnosis

BCR-ABL1 transcript levels at diagnosis varied in a wide range (0.0114–598.64%) with a median of 43.20%. In order to determine whether the transcript level at diagnosis could be used as a prognostic indicator for disease progression, we compared patients who achieved ≤10% BCR-ABL1 at 3 months (*n* = 281) and those who did not (*n* = 131). The BCR-ABL1 median ratio at diagnosis was 42.80% in the first group and 44.77% in the second, and no significant difference was found between them. Besides, no prognostic cutoff could be identified for BCR-ABL1 transcript levels at diagnosis (Table 2).

### Prognostic significance of 3-month 10% BCR-ABL1 cutoff

**Fig. 1:**
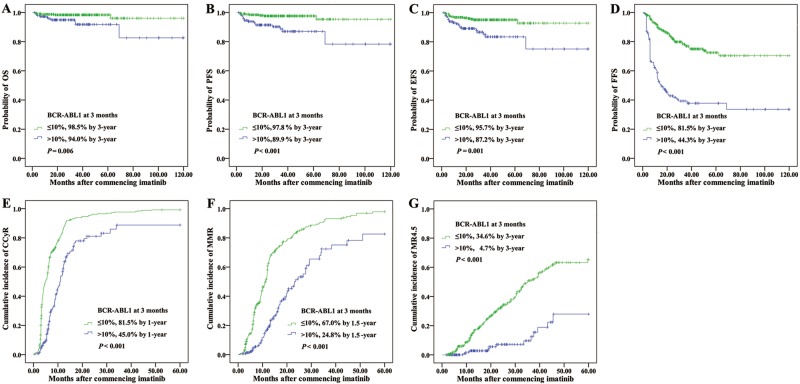
Outcomes according to 3-month BCR-ABL1 value. **a** OS, **b** PFS, **c** EFS, **d** FFS, **e** CCyR, **f** MMR, and **g** MR^4.5^

Of the total 476 patients, 473 had a BCR-ABL1 assessment at 3 months and median values was 3.55% (range 0.0025–141.62%). It was recently demonstrated that the persistence of BCR-ABL1 transcript levels >10% at 3 months identified a group of high-risk patients that would benefit from treatment optimization^5,6^. As expected, the 324 patients (68% of all patients) with BCR-ABL1 values ≤10% at 3 months had significantly better outcome than those with >10% (*n* = 149, 31% of all patients). The outcomes comparing ≤10% vs >10% BCR-ABL1 were as follows: 3-year OS, 98.5% vs 94.0%, *P* = 0.006; 3-year PFS, 97.8% vs 89.9%, *P* < 0.001; 3-year EFS, 95.7% vs 87.2%, *P* = 0.001; 3-year FFS, 81.5% vs 44.3%, *P* < 0.001; 1-year CCyR, 81.5% vs 45.0%, *P* < 0.001; 1.5-year MMR, 67.0% vs 24.8%, *P* < 0.001; and 3-year MR^4.5^, 34.6% vs 4.7%, *P* < 0.001 (Fig. 1).

Notwithstanding the usefulness of 3-month 10% BCR-ABL1 values for outcome prediction, there was a considerable number of patients with BCR-ABL1 >10% who did not fail to therapy and some subsequently achieve satisfactory outcomes despite being initially categorized as a poor responder at 3 months (Table 3). Sixty-three of 149 patients (42.3%) with >10% BCR-ABL1 achieved an MMR, which was maintained in 60 patients (95.2%) at the recent follow-up (median 18 months; range, 3–98.9 months). Furthermore, those patients with BCR-ABL1 ≤10% were classified as a good responder at 3 months; however, not all patients within this subgroup reached an optimal response and eventually favorable outcome (Table 3). At the first 18-month follow-up, 107 of 324 patients with ≤10% BCR-ABL1 had not achieved an MMR, which was sustained in 72 patients (67.3%) during the succeeding 18 months follow-up. Of those patients with ≤10% at 3 months, 2 were already dead and 5 subsequently progressed, as well as 11 lost CHR and MCyR, which occurred before 24 months. Thus, 3-month 10% BCR-ABL1 cutoff could not fully predict treatment response and ultimately clinical outcome, which urgently needs to seek the novel indicators for more precise and individual prognosis evaluation.

**Table 3:**
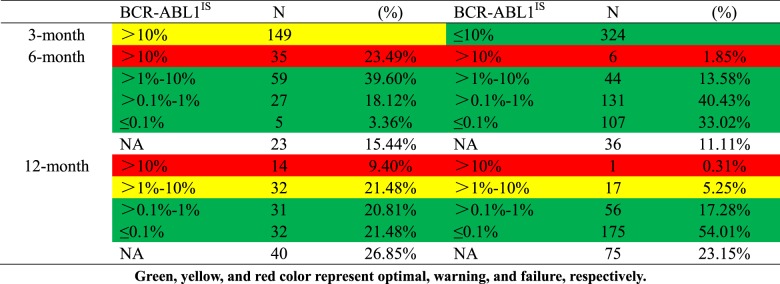
Distribution of *BCR-ABL1* values at 3, 6, and 12 months with 0.1% IS, 1% IS, and 10% IS as cutoff values

### BCR-ABL1 log reduction between diagnosis and 3 months

We thereafter investigated whether another cutoff point could predict long-term achievement of molecular response and outcome in our cohort. Based on the fact that the initial tumor burden might reflect different extents of an ongoing disease^18,19^, our study focused on the kinetics of the BCR-ABL1 decline from the individual patient baseline point to the 3-month values. We observed that some patients had very little or no decline, whereas others had more than a 50-fold reduction. Moreover, patients with the same BCR-ABL1 values at 3 months had better outcomes if their baseline values were higher. These findings demonstrated that the velocity of BCR-ABL1 decline may be critical for prognosis.

We therefore estimated the log reduction of 3-month BCR-ABL1 values from the individual baseline values and found that lower log reduction indicated a slow or no decline of BCR-ABL1, suggesting unsatisfactory outcomes. Of 412 evaluable patients, the median reduction was a 10.23-time decrease, corresponding to a 1.01 log reduction. Using ROC analysis, the optimal log reduction thresholds for discriminating between outcomes were as follows: OS, 0.61 (AUC, 0.727; 95% CI, 0.582–0.872); PFS, 0.61 (AUC, 0.734; 95% CI, 0.628–0.841); EFS, 0.71 (AUC, 0.713; 95% CI, 0.626–0.801); FFS, 0.91 (AUC, 0.745; 95% CI, 0.693–0.798); CCyR, 0.68 (AUC, 0.782; 95% CI, 0.729–0.836); MMR, 0.91 (AUC, 0.759; 95% CI, 0.711–0.807); and MR^4.5^, 1.18 (AUC, 0.742; 95% CI, 0.693–0.792). Of the highly relevant outcomes OS and PFS, we identified the optimal value of 0.61 log reduction at 3 months as the best predictive cutoff for outcome evaluation.

**Fig. 2:**
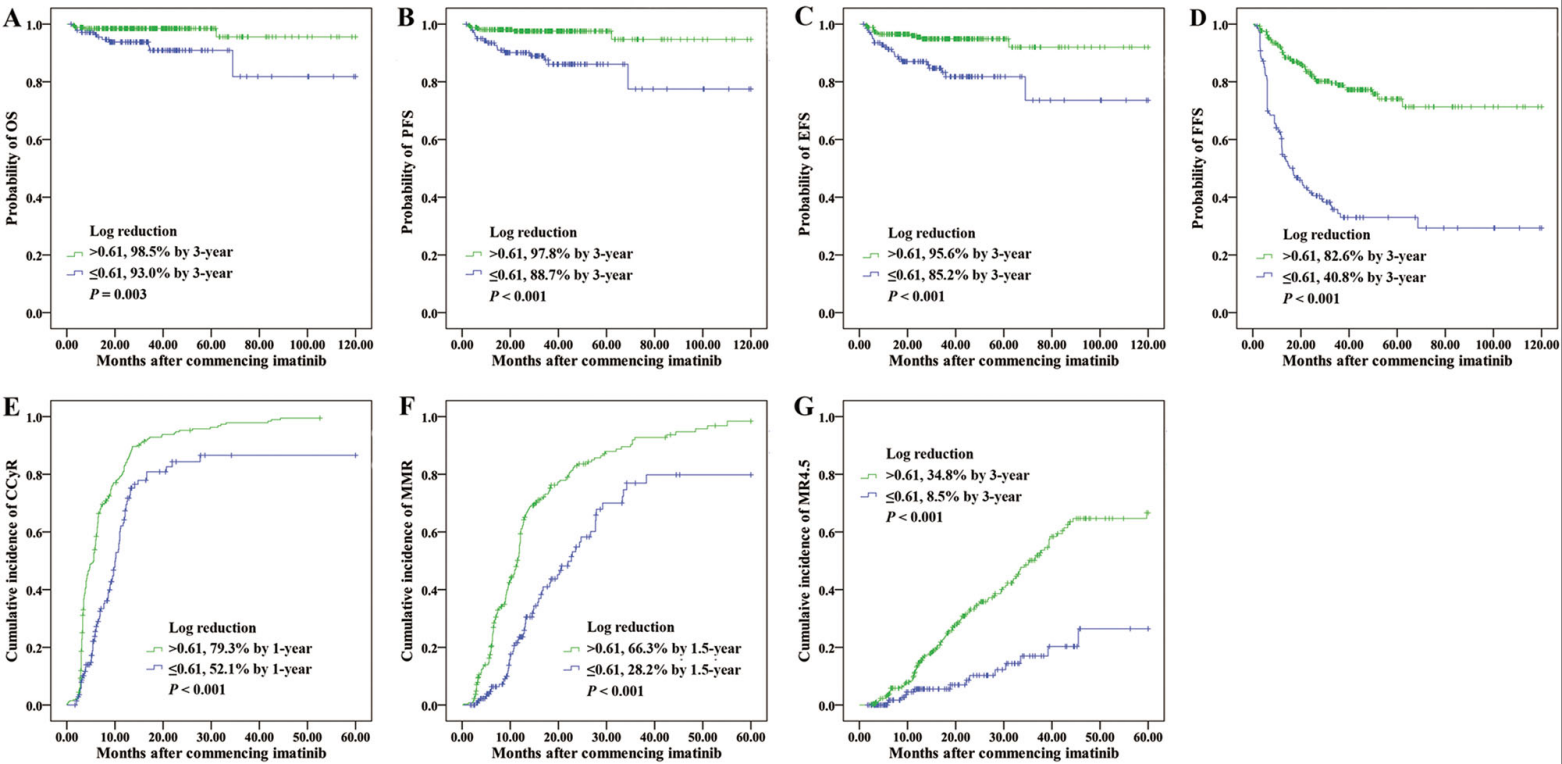
Outcomes according to BCR-ABL1 log reduction. **a** OS, **b** PFS, **c** EFS, **d** FFS, **e** CCyR, **f** MMR, and **g** MR^4.5^

Those patients with BCR-ABL1 >0.61 log reduction (270/412 evaluable patients, 66%) had significantly superior outcomes compared with the 142 of 412 patients (34%) where the log reduction was ≤0.61 (3-year OS, 98.5% vs 93.0%, *P* = 0.003; 3-year PFS, 97.8% vs 88.7%, *P* < 0.001; 3-year EFS, 95.6% vs 85.2%, *P* < 0.001; 3-year FFS, 82.6% vs 40.8%, *P* < 0.001; 1-year CCyR, 79.3% vs 52.1%, *P* < 0.001; 1.5-year MMR, 66.3% vs 28.2%, *P* < 0.001; and 3-year MR4.5, 34.8% vs 8.5%, *P* < 0.001, Fig. 2). Similarly with 3-month 10% BCR-ABL1, the 0.61 log reduction cutoff in our study was demonstrated to be another effective predictor of survival (Table 2).

**Fig. 3:**
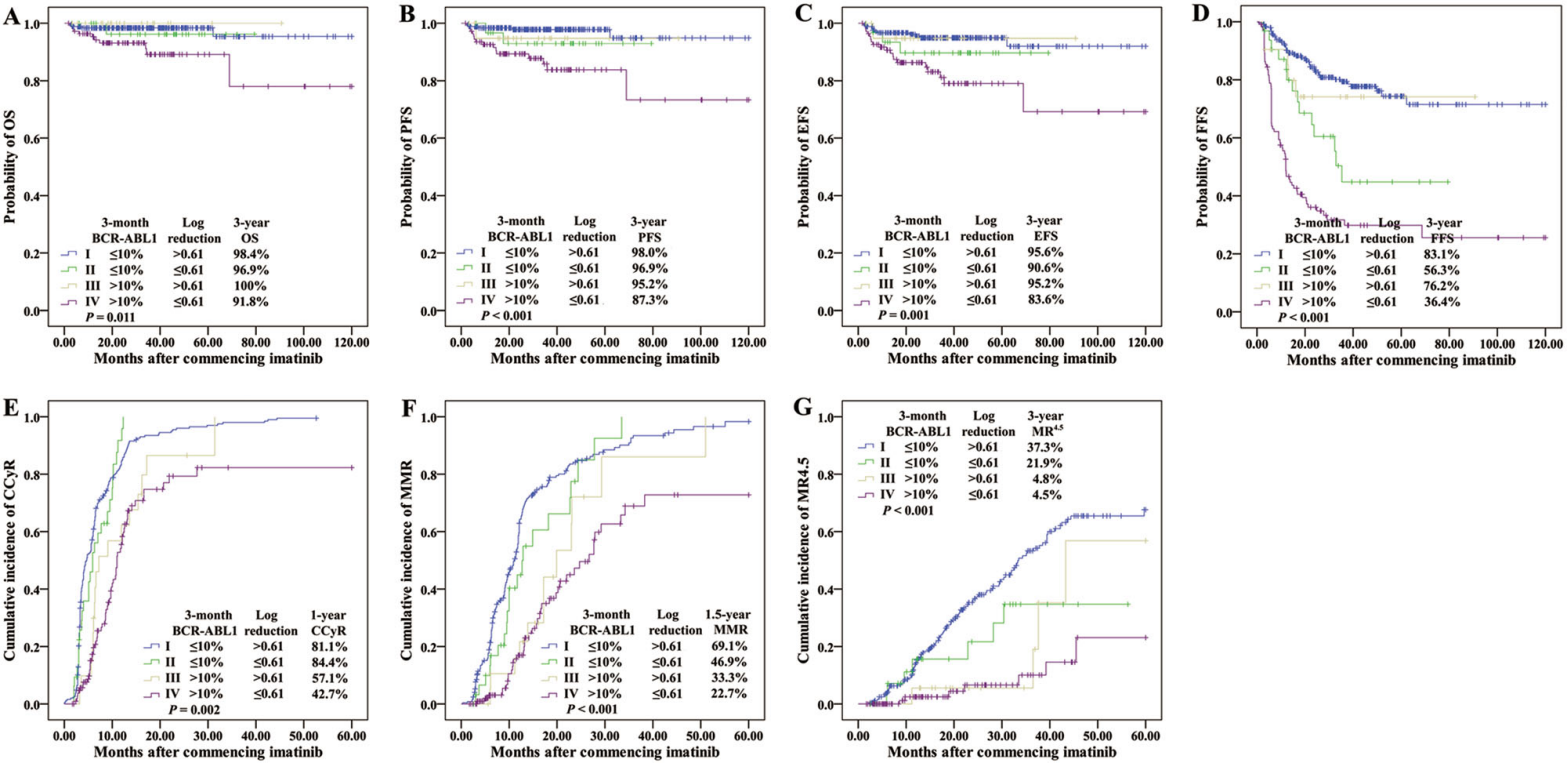
Outcomes according to both 3-month BCR-ABL1 value and log reduction. **a** OS, **b** PFS, **c** EFS, **d** FFS, **e** CCyR, **f** MMR, and **g** MR^4.5^

Of note, we also evaluated the discriminatory power for outcome prediction of the 0.61 log reduction combined with 10% cutoff at 3 months. Four groups were defined as follows: group I included those who achieved BCR-ABL1 transcript levels ≤10% and >0.61 log reduction both at 3 months. Groups II included those who achieved BCR-ABL1 transcript levels ≤10% and ≤0.61 log reduction both at 3 months. Groups III included those who failed to achieve an EMR (BCR-ABL1 >10%) and >0.61 log reduction both at 3 months. Groups IV included those who failed to achieve an EMR and ≤0.61 log reduction both at 3 months. When outcomes were compared among these four groups, statistical differences were noted in OS, PFS, EFS, and FFS at 3 years as well as CCyR at 1 year, MMR at 1.5 years and MR^4.5^ at 3 years (Fig. 3 and Supplementary Table 1). Consistently, subgroup analyses comparing Group I vs IV showed differences in OS (98.4% vs 91.8%, *P* = 0.005), PFS (98.0% vs 87.3%, *P* < 0.001), EFS (95.6% vs 83.6%, *P* < 0.001), FFS (83.1% vs 36.4%, *P* < 0.001), CCyR (81.1% vs 42.7%, *P* < 0.001), MMR (69.1% vs 22.7%, *P* < 0.001) and MR^4.5^ (37.3% vs 4.5%, *P* < 0.001) in favor of Group I. Comparing Groups I and II, Group II showed inferior FFS (83.1% vs 56.3% at 3 years, *P* = 0.001), but no difference in OS (98.4% vs 96.9%, *P* = 0.557), PFS (98.0% vs 96.9%, *P* = 0.181) and EFS (95.6% vs 90.6%, *P* = 0.026). However, when subgroup analyses were restricted to Groups I and III, there were no differences in OS (98.4% vs 100%, *P* = 0.706), PFS (98.0% vs 95.2%, *P* = 0.405), EFS (95.6% vs 95.2%, *P* = 0.885) and FFS (83.1% vs 76.2%, *P* = 0.338) between those two groups. Together, the 110 patients (26.7%) with BCR-ABL1 >10% and ≤0.61 log reduction at 3 months were identified to have the poorest survival.

Importantly, multivariate regression analysis demonstrated that a BCR-ABL1 log reduction calculated at 3 months of 0.61 was the only variable that significantly predicted for OS (HR, 6.539; 95% CI, 1.512–28.283; *P* = 0.012).

### BCR-ABL1 halving time

We investigated whether the patient with the most favorable outcome could be identified at an earlier time point. *BCR-ABL1* halving time was examined for this purpose. As illustrated in Supplementary Fig. 1, BCR-ABL1 values decline from baseline is exponential, demonstrating the halving time calculation is valid. We performed ROC analysis to determine the optimal halving time thresholds to predict outcome: OS, 44 days (AUC, 0.731; 95% CI, 0.585–0.877); PFS, 44 days (AUC, 0.736; 95% CI, 0.629–0.842); EFS, 39 days (AUC, 0.715; 95% CI, 0.627–0.803); FFS, 30 days (AUC, 0.744; 95% CI, 0.691–0.797); MMR, 28 days (AUC, 0.766; 95% CI, 0.721–0.811); and MR^4.5^, 22 days (AUC, 0.760; 95% CI, 0.710–0.809). Since this predictive marker could contribute to assess early response to first-line TKI and relieve the potentially psychological burden of patients, we selected the optimal MR^4.5^ halving time of 22 days for further outcome prediction in favor of sensitivity.

**Fig. 4:**
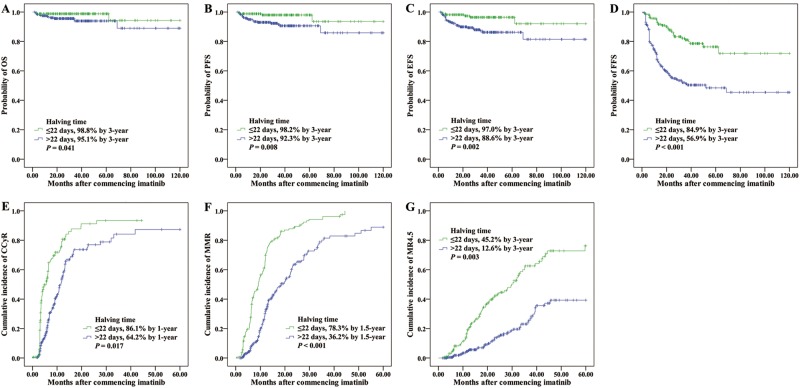
Outcomes according to BCR-ABL1 halving time. **a** OS, **b** PFS, **c** EFS, **d** FFS, **e** CCyR, **f** MMR, and **g** MR^4.5^

Of the 166 patients (40.3%) with BCR-ABL1 halving time ≤22 days, only one patients did not achieved ≤10% BCR-ABL1 at 3 months, whereas was still survival without progression during 5-year follow-up. Patients with halving time ≤22 days had significantly superior outcomes compared with patients whose BCR-ABL1 values did not halve by 22 days (*n* = 246; 3-year OS, 98.8% vs 95.1%, *P* = 0.041; 3-year PFS, 98.2% vs 92.3%, *P* = 0.008; 3-year EFS, 97.0% vs 88.6%, *P* = 0.002; 3-year FFS, 84.9% vs 56.9%, *P* < 0.001; and 1-year CCyR, 86.1% vs 64.2%, *P* = 0.017; 1.5-year MMR, 78.3% vs 36.2%, *P* < 0.001; 3-year MR^4.5^, 45.2% vs 12.6%, *P* = 0.003, Fig. 4 and Table 2). Therefore, BCR-ABL1 halving time ≤22 days could be recognized as a critical prognostic discriminator for the best prognosis patients.

**Fig. 5:**
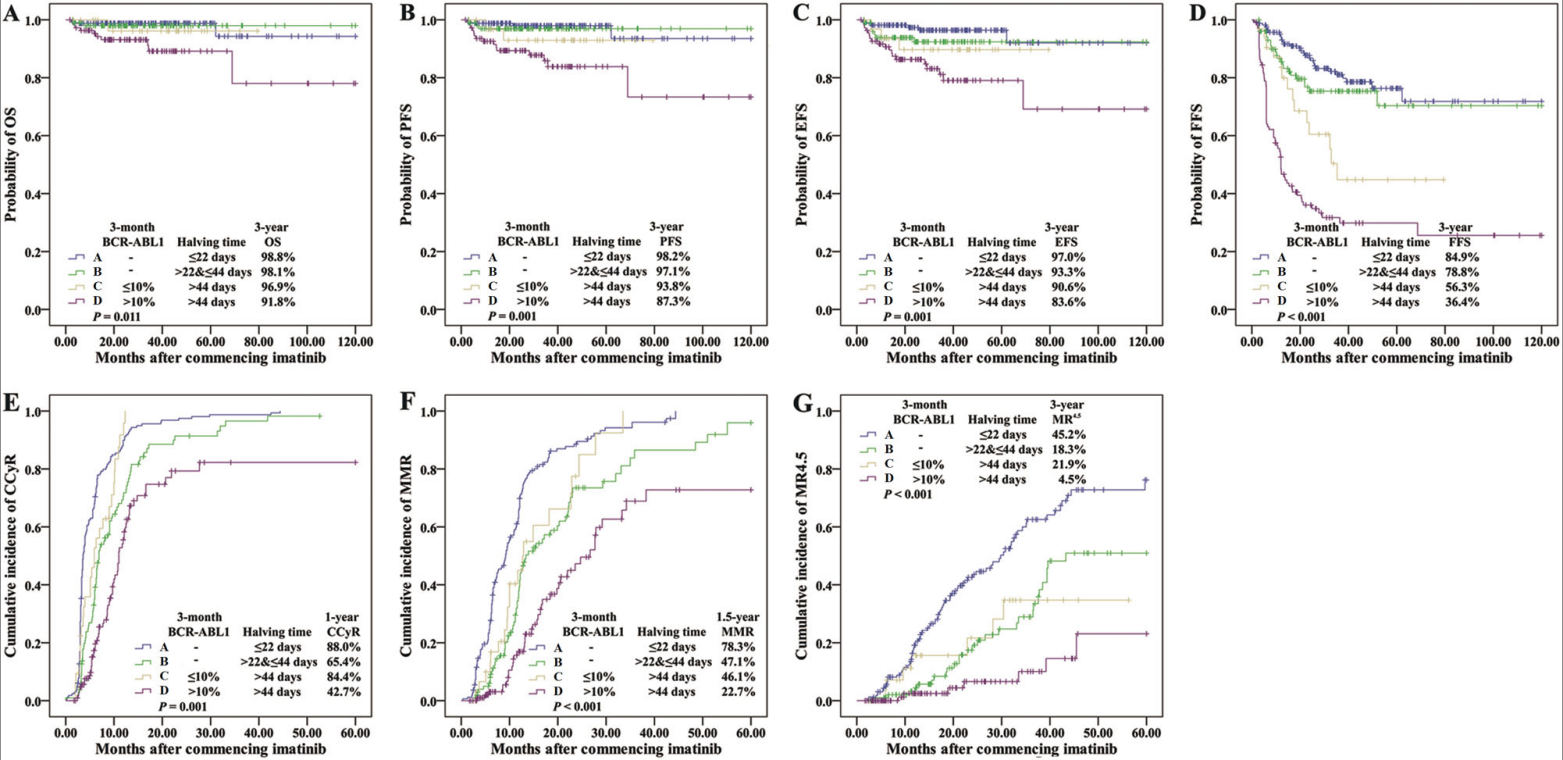
Outcomes according to both 3-month BCR-ABL1 value and halving time. **a** OS, **b** PFS, **c** EFS, **d** FFS, **e** CCyR, **f** MMR, and **g** MR^4.5^

Our above study illustrated that among the patients with >10% at 3 months, minimal or no decline from the baseline BCR-ABL1 values indicated the high risk of unfavorable outcome. In order to improve response and thereby minimize exposure to risk over time, we would like to find a better early indicator of the poorest-risk patients, who could benefit from alternative treatment as early as possible. Data showed that all outcomes were significantly inferior for the 142 patients (34.5%) with halving time >44 days (*P* < 0.01, equal to log reduction ≤0.61). More importantly, among the 31.8% of evaluable patients in our cohort with BCR-ABL1 >10% at 3 months, BCR-ABL1 halving time >44 days was statistically associated with the worst outcomes (*n* = 110; 3-year OS 91.8%, PFS 87.3%, EFS 83.6%, FFS 36.4% and MR^4.5^ 4.5%; Group D in Supplementary Table 2), demonstrating that lack of a BCR-ABL1 decline or a slow decline from baseline conveyed the highest risk of treatment failure, progression, and death. The comparison of clinical outcome and response to imatinib therapy among the group A-D were presented in Fig. 5 with respect to halving time (22 and 44 days) and 3-month 10% BCR-ABL1 values.

## Discussion

In the present study, we confirmed the strong predictive value of three EMR: 3-month BCR-ABL1 values, log reduction, and halving time of BCR-ABL1 levels between diagnosis and 3 months. Our results suggested that patients with BCR-ABL1 values on a constant and rapid downward trajectory had a high chance of reaching an optimal response and ultimately achieving a favorable outcome. However, among the patients with >10% at 3 months, lack of a BCR-ABL1 decline or a slow decline from baseline conveyed the highest risk of treatment failure, progression, and death.

High BCR-ABL transcript levels before treatment were reported to be associated with inferior probabilities of optimal response^18,20^. In contrast, recent studies demonstrated that BCR-ABL values at diagnosis cannot predict the achievement of molecular response and patient survival^10,21^. Consistent with the absence of prognostic value of pre-treatment transcript levels, we did not found any predictive cutoff with regard to survival. This may be because the emergence of additional mutations was rare at diagnosis, and resulted from the outgrowth of the BCR-ABL1-positive clone, high BCR-ABL values were elevated as the disease progressed.

In the current era of various TKIs, the early selection of patients requiring alternative treatment is critical. Previous studies demonstrated the prognostic value of 3-month BCR-ABL1 transcript levels^5,6^. Marin et al. identified cutoffs in the 3-month transcript levels (9.84%) that are predictors for OS using ROC curves^5^. Hanfstein et al. reported the predictive role of BCR-ABL1 transcript levels of 10% at 3 months for PFS and OS^6^. Consistently, our analysis verified that 3-month EMR was also significantly associated with higher cytogenetic and molecular response rates. The estimation of 3-month EMR predicted higher survivals with a decreased risk of progression.

It is no surprising that the prediction based on the rate of reduction seems to be more precise than the use of isolated 3-month BCR-ABL1 values^10,11,22^. In our study, the molecular 0.61-log reduction landmark (equal to 44 days-halving time) yields a relatively small *P* value and high Hazard ratio when compared with 3-month 10% BCR-ABL1 values, indicating a more precise prediction. Hanfstein et al. found that the lack of a 0.46-log reduction of BCR-ABL1 transcripts at 3 months was a discriminator of patients at risk for disease progression^10^. Branford et al. emphasized that the rate of BCR-ABL1 decline as assessed by BCR-ABL1 halving time (76 days) was a critical predictor for very poor outcomes among patients with BCR-ABL1 >10% at 3 months^11^. Thus, the velocity of early transcript elimination in the Chinese population accordingly appeared to be earlier than those in the west, possibly due to the relatively higher imatinib plasma levels in the Chinese patients^14,15^. Previous reports showed that imatinib trough level on day 29 were significantly associated with an achievement of 3-month EMR^9,23^.

Since prognostic information available even as early as 3 months may be too late for effective intervention in patients who experience early transformation^24^, we evaluated this optimal time point as being 22 days after the start of treatment in our cohort. Patients with a more rapid decline (BCR-ABL1 halving time ≤22 days) had a higher chance of ultimately achieving MMR and therefore a better prognosis. Therefore, BCR-ABL1 halving time ≤22 days was identified as a better early cutoff for patients with the most favorable outcome.

Additionally, our study highlights the importance of performing molecular analysis periodically to assess the BCR-ABL1 decline over the critical first 3 months, which may provide a cost-effective process for the better identification of patients for whom the risks and potential additional drug costs of therapy change are justified. Therefore, in a near future the survival prediction of each patient to TKI therapy will not only use the raw transcript levels but also different time-dependent variables assessing the BCR-ABL1 kinetics which are predictive of future molecular response and survival. Standardization of halving time or velocity of reduction will likely help establish more stringent recommendation and modify current clinical practices.

In conclusion, our study demonstrated that patients with CML-CP treated with imatinib can be stratified according to the BCR-ABL1 kinetics and that this stratification might contribute to the timing and necessity of therapeutic intervention. Because TKI therapy might have regional effects and might reflect distinct patient clinical profiles, we hope to validate these findings by studying larger cohorts in each region in the near future.

## Electronic supplementary material

Supplementary Figure 1 Legend

Supplementary Figure 1

Supplementary Table 1

Supplementary Table 2
