# Supplementary figures and images for: Early BCR-ABL1 decline in imatinib-treated patients with chronic myeloid leukemia: results from a multicenter study of the Chinese CML alliance
Source: Blood Cancer J. 2018 Jun 15;8(7):61. doi: 10.1038/s41408-018-0093-4 (PMC6006175; doi:10.1038/s41408-018-0093-4)

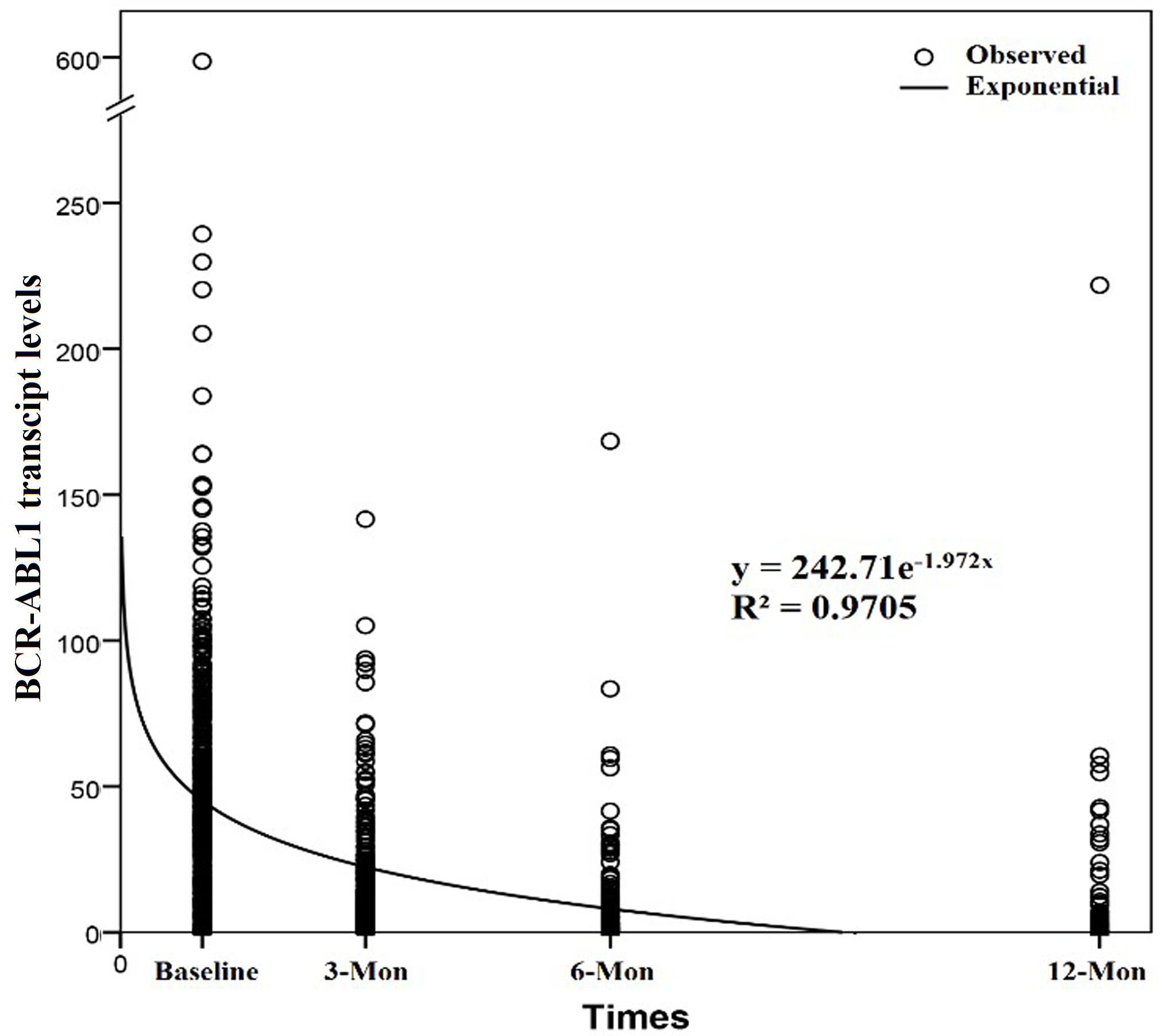

Supplement: Supplementary file 2 — Supplementary Figure 1 [file 41408_2018_93_MOESM2_ESM.tif]
