# Supplementary material for: Early BCR-ABL1 decline in imatinib-treated patients with chronic myeloid leukemia: results from a multicenter study of the Chinese CML alliance
Source: Blood Cancer J. 2018 Jun 15;8(7):61. doi: 10.1038/s41408-018-0093-4 (PMC6006175; doi:10.1038/s41408-018-0093-4)
Supplement: Supplementary file 3 — Supplementary Table 1 [file 41408_2018_93_MOESM3_ESM.docx]

**Supplementary Table 1 Outcomes of imatinib therapy according to the Groups by *BCR-ABL1* values at 3 months and log reduction**

| Group | 3-month | Log reduction | No. | OS (%) | PFS (%) | EFS (%) | FFS (%) | CCyR (%) | MMR (%) | MR^4.5^ (%) |
| --- | --- | --- | --- | --- | --- | --- | --- | --- | --- | --- |
| I | ≤10% | >0.61 | 249 | 98.4 | 98.0 | 95.6 | 83.1 | 81.1 | 69.1 | 37.3 |
| II | ≤10% | ≤0.61 | 32 | 96.9 | 96.9 | 90.6 | 56.3 | 84.4 | 46.9 | 21.9 |
| III | >10% | >0.61 | 21 | 100 | 95.2 | 95.2 | 76.2 | 57.1 | 33.3 | 4.8 |
| IV | >10% | ≤0.61 | 110 | 91.8 | 87.3 | 83.6 | 36.4 | 42.7 | 22.7 | 4.5 |
| *P* value between I vs II | | | | 0.557 | 0.181 | 0.260 | 0.001 | 0.950 | 0.093 | 0.277 |
| HR/RR between I vs II | | | | 0.518 | 0.327 | 0.480 | 0.346 | 1.103 | 1.571 | 1.532 |
| *P* value between I vs III | | | | 0.706 | 0.405 | 0.885 | 0.338 | 0.008 | 0.003 | 0.030 |
| HR/RR between I vs III | | | | 22.689 | 0.401 | 0.859 | 0.636 | 2.194 | 3.134 | 8.902 |
| *P* value between I vs IV | | | | 0.005 | <0.001 | <0.001 | <0.001 | <0.001 | <0.001 | <0.001 |
| HR/RR between I vs IV | | | | 0.187 | 0.149 | 0.253 | 0.176 | 2.954 | 4.070 | 7.516 |

**Abbreviations: HR, Hazard ratio for OS, PFS, EFS and FFS; RR, Relative risk for CCyR, MMR and MR^4.5^.**
