# Supplementary material for: Early BCR-ABL1 decline in imatinib-treated patients with chronic myeloid leukemia: results from a multicenter study of the Chinese CML alliance
Source: Blood Cancer J. 2018 Jun 15;8(7):61. doi: 10.1038/s41408-018-0093-4 (PMC6006175; doi:10.1038/s41408-018-0093-4)
Supplement: Supplementary file 4 — Supplementary Table 2 [file 41408_2018_93_MOESM4_ESM.docx]

**Supplementary Table 2 Outcomes of imatinib therapy according to the groups by *BCR-ABL1* values at 3 months and halving time**

| Group | 3-month | Halving time | No. | OS (%) | PFS (%) | EFS (%) | FFS (%) | CCyR (%) | MMR (%) | MR^4.5^ (%) |
| --- | --- | --- | --- | --- | --- | --- | --- | --- | --- | --- |
| A | - | ≤22 days | 166 | 98.8 | 98.2 | 97.0 | 84.9 | 88.0 | 78.3 | 45.2 |
| B | - | >22 days, ≤44 days | 104 | 98.1 | 97.1 | 93.3 | 78.8 | 65.4 | 47.1 | 18.3 |
| C | ≤10% | >44 days | 32 | 96.9 | 93.8 | 90.6 | 56.3 | 84.4 | 46.1 | 21.9 |
| D | >10% | >44 days | 110 | 91.8 | 87.3 | 83.6 | 36.4 | 42.7 | 22.7 | 4.5 |
| *P* value between A vs B | | | | 0.632 | 0.549 | 0.146 | 0.120 | <0.001 | <0.001 | <0.001 |
| HR/RR between A vs B | | | | 0.619 | 0.613 | 0.427 | 0.634 | 2.135 | 2.345 | 2.995 |
| *P* value between A vs C | | | | 0.438 | 0.178 | 0.123 | <0.001 | 0.264 | 0.012 | 0.088 |
| HR/RR between A vs C | | | | 0.387 | 0.293 | 0.324 | 0.294 | 1.265 | 1.983 | 1.965 |
| *P* value between A vs D | | | | 0.011 | 0.001 | <0.001 | <0.001 | <0.001 | <0.001 | <0.001 |
| HR/RR between A vs D | | | | 0.138 | 0.132 | 0.168 | 0.152 | 3.723 | 5.132 | 9.689 |

**Abbreviations: HR, Hazard ratio for OS, PFS, EFS and FFS; RR, Relative risk for CCyR, MMR and MR^4.5^.**
